# Cobalamin and iron deficiency still presents a challenge in hereditary hemorrhagic telangiectasia

**DOI:** 10.1038/s41598-025-13911-6

**Published:** 2025-08-04

**Authors:** Marie Carolin Schleupner, Alexander Röth, Luise Adam, Nadia Sadok, Felicia Toppe, Antonia Klara Lakomek, Sami Wainwright, Noemi Voss, Lukas Boosfeld, Christina Kaiser, Julia Garvert, Stephan Lang, Urban Geisthoff, Freya Droege

**Affiliations:** 1 VASCERN HHT Reference Centre, Department of Otorhinolaryngology, Head and Neck Surgery, University Hospital Essen, Hufelandstraße 55, 45147 Essen, Germany; 2 Clinic for Internal Medicine and Hematology, University Hospital Essen, Essen, Germany; 3 Institute of Primary Health Care (BIHAM), University of Bern, Bern, Switzerland; 4 Division of Internal Medicine, Angiology, Cantonal Hospital of Schaffhausen, Schaffhausen, Switzerland; 5 VASCERN HHT Reference Centre, Department of Otorhinolaryngology, Head and Neck Surgery, University Hospital Marburg, Philipps-Universität Marburg, Baldingerstrasse, 35043 Marburg, Germany

**Keywords:** HHT, Anemia, Cobalamin deficiency, Iron deficiency, Transfusions, Outcomes research, Medical research, Epidemiology

## Abstract

Hereditary hemorrhagic telangiectasia (HHT) leads to fragile blood vessels, causing frequent bleeding and anemia. Treatment mainly addresses iron levels and substitution. Although cobalamin (vitamin B12) is routinely tested in chronic anemia, its role in HHT has not been studied until now. This study examined its prevalence and related symptoms in HHT patients. Data from HHT patients treated between July 2019 and November 2022 were analyzed. Patients with cobalamin levels under 400 pg/ml underwent further testing and questioning. Among 155 patients, 42% were anemic, and 45% had cobalamin levels below 400 pg/ml. However, only 7 patients had a confirmed deficiency. Still, many reported symptoms commonly but not specifically linked to cobalamin deficiency, even with low-normal levels. Gastrointestinal lesions were significantly associated with these low-normal values (*p* = 0.027). Furthermore, 59% of patients had iron deficiency, and 67% were not receiving iron therapy at the time of their visit. This study is the first to show that true cobalamin deficiency is not more common in HHT than in the general population, though low-normal levels are frequent. In case of unexplained symptoms, evaluating cobalamin levels should be taken into consideration.

**Supplementary Information:**

The online version contains supplementary material available at 10.1038/s41598-025-13911-6.

## Introduction

In patients with hereditary hemorrhagic telangiectasia (HHT), mutations in the transforming growth factor beta signaling pathway lead to systemic vascular malformations. The disease is diagnosed when at least 3 out of the 4 Curaçao criteria apply (history of HHT in patients’ families, recurrent epistaxis, presence of telangiectasia and arteriovenous malformations in different organs especially in the liver, lungs, gastrointestinal tract or the brain) or when one of the mutations known to cause HHT is found in molecular genetic analysis^1–4^. Patients with HHT often suffer from recurring episodes of bleeding (especially epistaxis and gastrointestinal bleeding) and may develop anemia^2^. Microcytic hypochromic anemia and iron deficiency are frequently observed^5,6^. While studies on non-HHT patients with chronic bleedings also describe concurring cobalamin (vitamin B12) deficiency^7^, to the best of our knowledge, cobalamin levels have not been described in HHT yet.

Cobalamin is essential in the upkeep of neural sheaths^8,9^ as well as the regeneration of cells^10^. Therefore, it also plays a crucial role in erythropoiesis^11^ and needs to be supplemented if not sufficiently available in the treatment of anemia. After oral consumption of food, cobalamin is conjugated with intrinsic factor released by gastric parietal cells to enable the uptake in the terminal ileum^12^. Only a small percentage of cobalamin (1–4%) is absorbed by intestinal tissue independently of intrinsic factor^13^. People following a vegan or strictly vegetarian diet can develop a deficiency as cobalamin is only found in animal products^14,15^. Patients with gastrointestinal diseases or a history of gastric surgery are likewise at risk of developing cobalamin deficiency when secretion of intrinsic factor is impaired^16^. Uptake is also reduced if pathologies of the terminal ileum exist, or it has been resected^17–19^. Due to the large storage capacity in the human body compared to the daily requirements (3–5 mg storage with daily recommended uptake and turnover of 2–4 µg^13,15^) cobalamin deficiency only occurs after years of insufficient uptake or increased loss. Symptoms include altered skin coloring (irregularities in coloring and pallor), glossitis, neurological impairment and macrocytic anemia^12,20^. The latter could, however, remain underdiagnosed when concurring with iron deficiency and normo- to microcytosis^7^.

Up to this point, no studies on the clinical relevance of cobalamin deficiency in HHT patients have been published. Cobalamin deficiency might be a significant issue in HHT patients mainly due to chronic bleeding anemia and gastrointestinal vascular malformations. In the following study, we aim to analyze the occurrence and clinical impact of cobalamin deficiency in patients with HHT.

## Materials and methods

We included HHT patients aged 18 and above presenting at the West German HHT Centre at the ENT department of the University Hospital Essen between July 2019 and November 2022 for routine check-ups, scheduled interventions or emergency consults. Inclusion required a confirmed HHT diagnosis, defined as either at least 3 of 4 Curaçao criteria or a positive genetic test for one of the known HHT-associated mutations^21^. Written informed consent was obtained from all included patients. Blood samples were taken as part of the routine laboratory workup including hemoglobin, erythrocyte parameters, cell counts, overall cobalamin, folic acid, C-reactive protein, liver enzymes and serum iron parameters (serum iron, transferrin concentration and saturation, soluble transferrin receptor, ferritin and haptoglobin). Patients were excluded if key laboratory parameters required for cobalamin status evaluation were missing.

Anemia was defined as hemoglobin (Hb) levels below 12 g/dl for women and below 13 g/dl for men^22^. Iron deficiency (regardless of hemoglobin levels) was classified by evaluation of mean corpuscular volume (MCV), ferritin, CRP (C-reactive protein) and soluble transferrin receptor serum levels according to current hematological guidelines^23^. To assess cobalamin deficiency more thoroughly when cobalamin levels were low or low-normal (up to 399 pg/ml^12^; reference in our laboratory 211 to 911 pg/ml), holotranscobalamin and methylmalonic acid were measured prospectively additionally from April 2022 onwards. Blood was then taken in an extra tube during the same visit and patients were questioned with special focus on symptoms of cobalamin deficiency. Possible comorbidities, dietary habits and medication associated with cobalamin deficiency were also recorded as possible confounders (see appendix for the exact questionnaire). Special attention was given to proton pump inhibitors (inhibition of gastric acid secretion could decrease cobalamin absorption as observed in select patients^24^) and metformin (whilst the exact pathomechanism remains unclear, reduced levels of cobalamin have been observed after long term therapy^25^).

We separately analyzed the subgroup of patients with iron supplementation (regardless of their iron status or hemoglobin levels) to assess its effects. Data on established iron therapy by the treating general practitioner were self-reported by the patients. Additional information pertaining to this study, especially concerning disease severity (using the epistaxis severity score (ESS)^26^ as surrogate parameter) and gastrointestinal angiodysplasia (confirmed by endoscopy) was taken from the patients’ medical records at our clinic.

Statistical analysis was performed using SPSS (Version 27, IBM Software). All variables were tested for normal distribution with the Shapiro-Wilk-Test. Correlations were evaluated using cross tables and χ^2^ (Chi square) analysis for categorical variables, Student’s t-Test for continuous variables if normally distributed and ANOVA, Kruskal-Wallis- or Mann-Whitney-U-Test if not. Correlations between continuous variables were examined with Spearman’s rank correlation and Pearson correlation. Graphs were created using Microsoft PowerPoint and Sankeymatic.

## Results

We included 155 patients with a mean (m) age of 54 years (± standard deviation (SD): 14 years) of which 65% were female (number of patients (n) = 101/155). All patients suffered from epistaxis (ESS (mean (m) ± SD): 5.3 ± 2.2; available for 132/155; 85% of the patients) and 32% of the patients (*n* = 50/155) reported gastrointestinal involvement (Table 1). 60% of those with gastrointestinal involvement had received gastrointestinal treatment (*n* = 30/50; coagulation or clipping; see Table 1 for patient characteristics). Higher ESS scores correlated significantly with lower hemoglobin levels (*p* < 0.001) and lower mean corpuscular volumes (*p* = 0.038) but not to serum ferritin levels (*p* = 0.602) or any other iron status parameter apart from soluble transferrin receptor levels which were markedly higher in patients with higher ESS scores (*p* < 0.001).

**Table 1:** Patient characteristics.

	All (*n* = 155)	Men (*n* = 54)	Women (*n* = 101)	*p*-value (χ^2^-test)
Age [years]	53.9 ± 14.3	54.4 ± 15.9	53.6 ± 13.4	0.470
Positive family history	138 (89%)	35 (65%)	93 (92%)	0.173
Genetic testing	18 (12%)	4 (7%)	14 (14%)	0.410
Epistaxis	155 (100%)	54 (100%)	101 (100%)	–/–
Telangiectasia	153 (99%)	53 (98%)	100 (99%)	0.651
Organ manifestations	100 (65%)	31 (57%)	69 (68%)	0.176
Brain	9 (6%)	5 (9%)	4 (4%)	0.179
Lungs	65 (42%)	20 (37%)	45 (45%)	0.366
Liver	24 (15%)	6 (11%)	18 (18%)	0.271
Gastrointestinal tract	50 (32%)	17 (31%)	33 (33%)	0.880

This table presents patients’ age and gender distribution, Curacao criteria and their distribution among genders. No significant differences between male and female patients were found. Since all patients suffered from epistaxis, no further analysis of this parameter was possible. All data given as mean ± standard deviation or absolute figures (n) and percentages.

### Evaluation of hemoglobin level

**Fig. 1:**
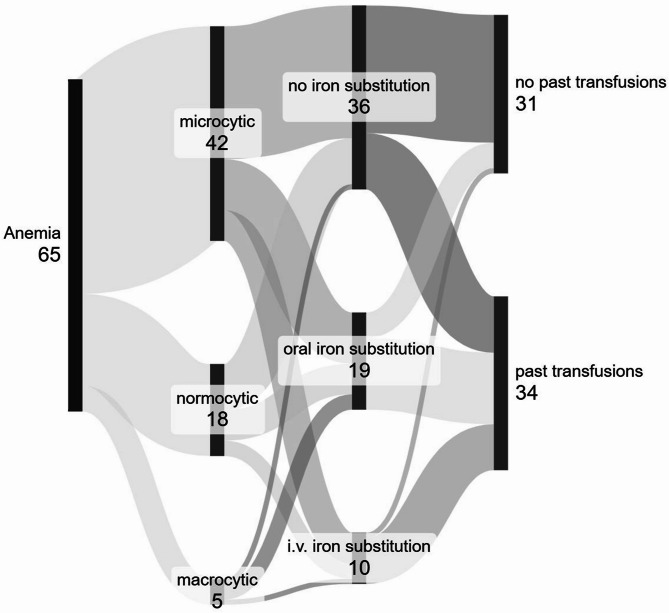
Overview of anemic patients with hereditary hemorrhagic telangiectasia. The subtype of anemia according to mean corpuscular volume and the treatment of anemia are shown (figure created with Sankeymatic). More than half of these patients (36/65, 55%) did not receive any iron supplementation. The large proportion of patients with past blood transfusions highlights the severity of anemia. i.v. = intravenous, proportion of patients and absolute numbers are shown.

Low hemoglobin levels were seen in 42% of the patients (*n* = 65/155; women below 12 g/dl (*n* = 39/101, 39%; men below 13 g/dl: *n* = 26/54, 48%; *p* = 0.252), thus fulfilling the WHO definition for anemia^27^. None of the patients were diagnosed with comorbidities typically leading to iron deficiency anemia such as thalassemia or sickle cell anemia at inclusion. Microcytic anemia was most common (*n* = 42/65 patients, 65%). However, it should be noted that five patients (*n* = 5/65; 8%) presented with macrocytic anemia which is typically seen in cobalamin deficiency. Only 29 patients (*n* = 29/65; 45%) regularly received iron substitutions at the time of their examination at the West German HHT Centre (only oral application: *n* = 19/65; 29%, intravenous application: *n* = 10/65; 15%). Most patients with low hemoglobin levels did not take any iron substitution when seen for the first time at the West German HHT Center (*n* = 36/65; 55%; see Fig. 1). Three of the patients with intravenous iron substitution (*n* = 3/10: 30%) also took oral iron medication, receiving intravenous therapy when oral therapy was insufficient. At some point in their lives, 34 out of 65 patients with low hemoglobin levels had received blood transfusions (m ± SD = 16 ± 76 erythrocyte concentrates with one patient reporting 600 past transfusions). Patients were significantly more likely to have received blood transfusions if they were still substituting iron (*n* = 14/19; 74% of the patients with oral substitution and 9/10; 90% with intravenous substitution compared to *n* = 11/36; 31% of the patients without current iron substitution; *p* < 0.001). In total, 55 patients (*n* = 55/155; 35%) had ever received blood transfusions. Six of these patients had already developed blood group antibodies (*n* = 6/55; 10%), 28 (*n* = 28/55; 51%) had none and no data concerning blood group antibodies was available for 21 patients (*n* = 21/55; 38%).

### Evaluation of iron status

**Fig. 2:**
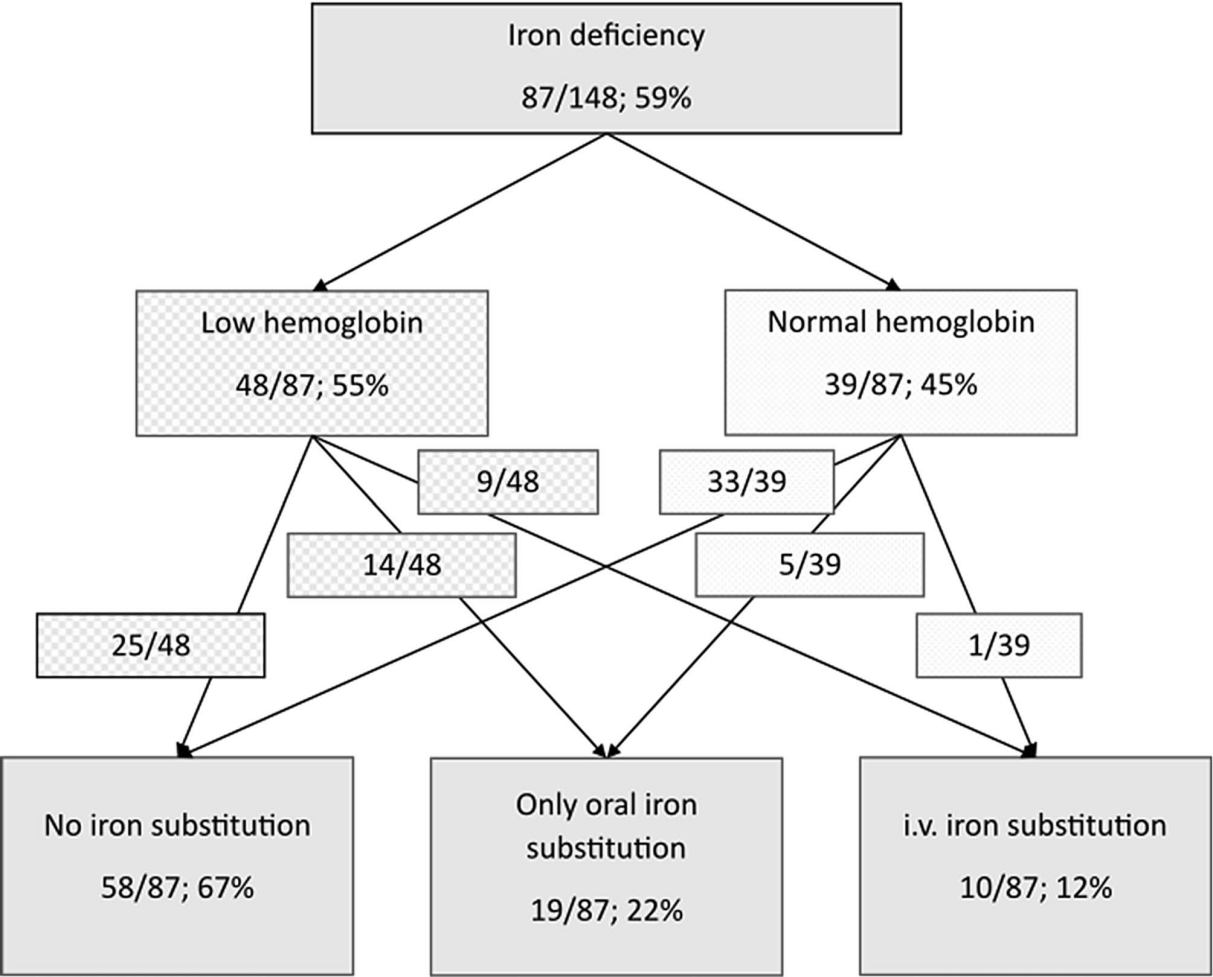
Iron deficiency in patients with hereditary hemorrhagic telangiectasia. An overview of patients with hereditary hemorrhagic telangiectasia and iron deficiency sorted by hemoglobin levels and iron substitution status. Most patients with iron deficiency and low hemoglobin levels (58/87; 67%) did not receive any kind of iron substitution at the time of this study. Iron deficiency was diagnosed based on further evaluation of mean corpuscular volume, ferritin, and C reactive protein according to hematological guidelines. Proportion of patients and percentage are shown. Low hemoglobin = below 12 g/dl for women, below 13 g/dl for men; Normal hemoglobin = ≥ 12 g/dl for women, ≥ 13 g/dl for men; i.v. = intravenous.

For seven patients (*n* = 7/155; 5%) no sufficient laboratory data was available to evaluate the iron status according to the criteria described above. 87 of the remaining 148 patients (*n* = 87/148; 59%) suffered from iron deficiency. Only 29 patients with a diagnosed iron deficiency reported regularly substituting iron (*n* = 29/87; 33%, only oral iron substitution: *n* = 19/87; 22%, intravenous iron substitution: *n* = 10/87; 12%). Most patients did not take any treatment for their iron deficiency (58/87; 67%) due to side effects, misperception of their symptoms related to iron deficiency or anemia or insufficient diagnostics prior to their visit at the West German HHT center. A detailed overview of this data is given in Fig. 2.

46 patients substituted iron (regardless of their iron status or hemoglobin levels; *n* = 46/155; 30%; oral: *n* = 36/155; 23% and intravenous: *n* = 10/155; 7%). A complete iron status analysis was available for 45 patients (*n* = 45/46; 97.8%). 16 patients (*n* = 16/45; 36%) showed no signs of iron deficiency in laboratory workup and 10 of these patients (*n* = 10/16; 63%) had normal hemoglobin levels (indicating a successful treatment). 29 patients (*n* = 29/45, 64%) with HHT still displayed overall iron deficiency despite their iron substitution of whom 23 patients (*n* = 23/29, 79%) were also anemic. 19 of these patients (19/29; 66%) received an oral treatment and 10 patients had intravenous iron substitutions (10/29; 35%).

### Cobalamin status

Average serum cobalamin levels were measured at 475.1 ± 249.6 pg/ml (m ± SD). One patient (1/155; 0.6%) had cobalamin levels below the reference range (201 pg/ml, reference range 211–911 pg/ml) and an additional number of 69 patients (*n* = 69/155; 45%) had levels between 211 and 399 pg/ml equaling lower normal levels. Men tended to display lower levels of cobalamin than women (503.1 ± 288.5 pg/ml in women and 422.6 ± 140.1 pg/ml in men, *p* = 0.068; Table 2). Even though it was expected that due to menstrual bleeding women might require more cobalamin for erythropoiesis, cobalamin levels did not differ significantly from men in the relevant age group (women and men up to 45 years old: *n* = 43/155, 27.7% and *p* = 0.688). Regarding age, both 45% of the patients older than 60 years (*n* = 26/58) and 45% of patients younger than 60 years (*n* = 44/97) showed low-normal cobalamin levels between 211 and 399 pg/ml. The same cobalamin levels (between 211 and 399 pg/ml) were observed more frequently in patients with anemia as defined by reduced hemoglobin (*n* = 35/65, 54%) than in patients with normal hemoglobin levels (*n* = 35/90, 39%, *p* = 0.065). Neither classification of anemia according to MCV as microcytic, normocytic or macrocytic nor the MCV itself was associated with cobalamin levels (*p* > 0.05 respectively). Cobalamin levels did not correlate with ferritin levels (*p* = 0.479), iron deficiency (*p* = 0.322) or ESS (*p* = 0.769). Iron deficiency (as defined by MCV, ferritin, CRP and soluble transferrin receptor serum levels) and low hemoglobin levels simultaneously were detected in 48 patients (*n* = 48/155; 31%). Of these, 24 patients (*n* = 24/48; 50%) also had cobalamin levels between 211 and 399 pg/ml. An overview of clinical characteristics and their correlation with cobalamin status is provided in Table 3.

**Table 2:** Laboratory results of patients with hereditary hemorrhagic telangiectasia.

	All			Male			Female			*p*-value
	[*n*]	[m ± SD]	[min-max]	[*n*]	[m ± SD]	[min-max]	[*n*]	[m ± SD]	[min-max]	
Leukocytes [/nl]	155	6.5 ± 2.0	2.8–14.7	54	6.5 ± 2.1	2.8–13.1	101	6.4 ± 2.0	3.2–14.7	0.943
Hemoglobin [g/dl]	155	12.3 ± 2.5	4.2–18.0	54	12.6 ± 3.0	6.4–18.0	101	12.1 ± 2.2	4.2–15.8	0.203
MCV [fl]	155	84.2 ± 7.7	61.3–108.1	54	82.3 ± 7.7	61.3–100.0	101	85.1 ± 7.5	66.1–108.1	**0.042**
MCH [pg]	155	27.4 ± 3.6	16.7–36.2	54	26.7 ± 3.9	18.0–31.9	101	27.8 ± 3.3	16.7–36.2	0.252
MCHC [g/dl]	155	32.5 ± 2.1	25.3–36.4	54	32.4 ± 2.4	27.3–35.9	101	32.6 ± 1.8	25.3–36.4	0.844
Hematocrit [l/l]	155	0.4 ± 0.1	0.2 − 0.5	54	0.4 ± 0.1	0.2 − 0.5	101	0.4 ± 0.1	0.2 − 0.5	0.099
Erythrocytes [/pl]	155	4.5 ± 0.7	2.2–5.9	54	4.7 ± 0.8	2.7 − 5.9	101	4.3 ± 0.6	2.2–5.5	**0.001**
Reticulocytes [%]	153	2.0 ± 1.4	0.4–10.4	54	2.1 ± 1.6	0.4–9.4	99	2.0 ± 1.3	0.7–10.4	0.982
Reticulocyte proliferation index	153	1.2 ± 0.6	0.1–3.0	54	1.3 ± 0.7	0.1–3.0	99	1.1 ± 0.5	0.2–2.9	0.193
Serum iron [µg/dl]	155	72.4 ± 66.4	7.0–484.0	54	78.9 ± 66.5	9.0–381.0	101	68.9 ± 66.4	7.0–484.0	0.281
Ferritin [µg/l]	154	58.9 ± 123.4	1.0–1.030.0	54	70.4 ± 133.2	2.0–779.0	100	52.8 ± 118.0	1.0–1.030.0	0.740
Transferrin [g/l]	129	2.8 ± 0.5	1.7 − 4.3	50	2.9 ± 0.5	1.8 − 4.2	79	2.8 ± 0.5	1.7 − 4.3	0.386
Soluble transferrin receptor [mg/l]	124	2.9 ± 2.5	0.9–17.9	50	3.4 ± 3.1	0.9–17.9	74	2.7 ± 2.0	0.9–14.1	0.240
Transferrin saturation [%]	129	19.0 ± 19.6	1.9–156.0	50	20.1 ± 16.7	1.9–81.9	79	18.2 ± 21.4	2.0–156.0	0.296
Folic acid [ng/ml]	144	13.3 ± 9.7	2.6–48.0	52	12.1 ± 8.9	3.6–48.0	92	14.0 ± 10.1	2.6–48.0	0.331
Haptoglobin [g/l]	42	1.1 ± 0.5	0.2–2.1	17	1.1 ± 0.5	0.6 − 2.0	25	1.1 ± 0.5	0.2–2.1	0.929
GOT [U/l]	142	25.7 ± 13.4	0.0–106.0	52	25.6 ± 10.9	9.0–55.0	90	25.7 ± 14.7	0.0–106.0	0.842
GPT [U/l]	142	25.0 ± 13.2	9.0–98.0	52	26.3 ± 13.7	9.0–80.0	90	24.2 ± 12.9	10.0–98.0	0.325
Creatinine [unit]	100	0.8 ± 0.2	0.3 − 1.6	34	0.9 ± 0.2	0.7 − 1.6	66	0.7 0.1	0.3 − 1.0	**< 0.001**
C-reactive protein [mg/dl]	136	0.5 ± 0.7	0.3–5.8	51	0.5 ± 0.7	0.3–4.0	85	0.5 ± 0.8	0.3–5.8	0.624
Cobalamin [pg/ml]	155	475.1 ± 249.6	201–2498	54	422.6 ± 140.1	201.0–910.0	101	503.1 ± 288.5	227.0–2.498.0	0.068
Holotranscobalamin [pmol/l]	23	79.7 ± 32.6	40.5–150.0	10	75.1 ± 37.1	44.3–150.0	13	83.2 ± 29.7	40.5–150.0	0.563
MMA [ng/ml]	23	27.1 ± 10.	11.3–44.8	10	31.1 ± 11.7	17.7–44.8	13	24.0 ± 7.7	11.3–40.3	0.257

An overview of all laboratory parameters relevant to the evaluation of anemia and cobalamin status for total population and by gender, p-values for gender differences were calculated using the Mann-Whitney U test. Women had significantly higher mean corpuscular volume (MCV, within the reference range) and lower concentration of erythrocytes (lower than the reference range) as well as lower creatinine serum levels. No significant differences were found for the other parameters.

MCH = mean corpuscular hemoglobin, MCHC = mean corpuscular hemoglobin concentration, GOT = glutamate-oxalacetate-transaminase, GPT = glutamate-pyruvate-transaminase, MMA = methylmalonic acid.

**Table 3:** Clinical characteristics and correlation with cobalamin status.

	Cobalamin < 400 pg/ml *n* = 70		Cobalamin ≥ 400 pg/ml *n* = 85		*p*-value
Gender	Women	Men	Women	Men	0.057
	40 (57.1%)	30 (42.9%)	61 (71.8%)	24 (28.2%)	
Age	< 60y	≥ 60y	< 60y	≥ 60y	0.949
	44 (62.9%)	26 (37.1%)	53 (62.4%)	32 (37.6%)	
Anemia	No	Yes	No	Yes	0.065
	35 (50%)	35 (50%)	55 (64.7%)	30 (35.3%)	
GI involvement	No	Yes	No	Yes	**0.027**
	41(58.6%)	29 (41.4%)	64 (75.3%)	21 (24.7%)	
Past erythrocyte transfusions	No	Yes	No	Yes	0.160
	41 (58.6%)	29 (41.4%)	59 (69.4%)	26 (30.6%)	
≥ 6 cobalamin deficiency symptoms*	No	Yes	No	Yes	0.827
	13 (30.2%)	30 (69.8%)	1 (25.0%)	3 (75.0%)	
≥ 1 neurological symptom*	No	Yes	No	Yes	0.330
	4 (9.3%)	39 (90.7%)	1 (25.0%)	3 (75.0%)	

Clinical characteristics which are otherwise known to be associated with cobalamin deficiency are listed along with their statistical correlation to cobalamin status. Only the presence of GI involvement stands out as statistically significant, seemingly predisposing patients to lower cobalamin levels. Cobalamin deficiency symptoms refer to the symptoms included in the questionnaire, listed in Table 4. Neurological symptoms were paresthesia or irregular sensations in hand and/or feet, dizziness, pathological gait and falling tendencies, weakness or tiredness, sleeping disturbances, difficulty in concentrating, impaired vision and impaired sensibility.

y = years; *data from questionnaire, only available for 47 patients.

**Fig. 3:**
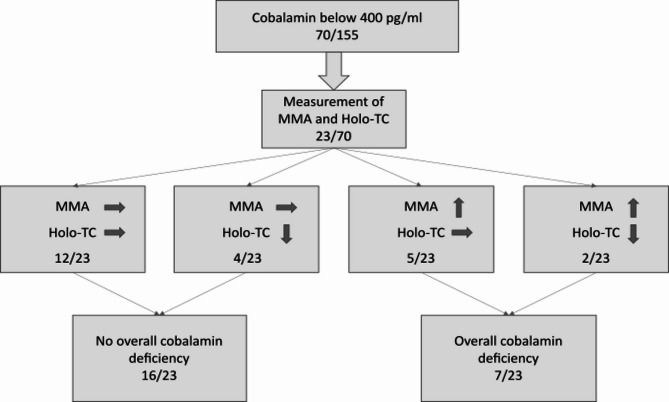
Diagnostic pathway for evaluation of functional cobalamin status and deficiency. Elevated MMA and reduced holotranscobalamin levels were detected in two patients (2/23; 9%) even though serum cobalamin levels were classified as normal but low at 284 and 337 pg/ml respectively. 5 patients (5/23; 22%) had elevated MMA levels but normal Holo-TC measurements and two of those patients had renal dysfunction, which may have caused falsely high Holo-TC readings. Overall deficiency was diagnosed when MMA levels were elevated even if Holo-TC levels were normal and ruled out when MMA levels were normal as MMA is considered the more specific parameter. MMA = methyl malonic acid, Holo-TC = holotranscobalamin, proportion of patients and percentage are shown.

For further evaluation of the overall cobalamin status, holotranscobalamin and methyl malonic acid (MMA) were measured in 23 patients with cobalamin levels below 400 pg/ml (*n* = 23/155; 15%). Regarding patients’ age, 11 patients of this group were aged 60 and above (*n* = 11/23; 48%). True cobalamin deficiency was diagnosed in seven HHT patients (*n* = 7/23; 30%, three of these seven patients (43%) also showed hemoglobin levels below the norm) as their MMA levels were elevated even though holotranscobalamin levels were still normal in five of these patients (*n* = 5/7; 71%). None of these seven patients (*n* = 0/7; 0%) had a macrocytic anemia. MMA was normal in the remaining 16 patients (*n* = 16/23; 70%) of which two showed lowered holotranscobalamin levels (*n* = 2/16; 13%) and a cobalamin deficiency was excluded. The evaluation pathway for our population is shown in Fig. 3. In patients over 60 years old elevated MMA levels were observed in five patients (*n* = 5/11; 45%) whereas only two of the patients younger than 60 years had elevated MMA levels indicating true cobalamin deficiency (*n* = 2/12; 17%).

Elevated cobalamin serum levels were detected in four patients (*n* = 4/155; 3%). In these patients no impairment of renal function or kidney deficiency were diagnosed and only one patient reported a cobalamin supplementation. Another two patients (*n* = 2/155; 1%) also received regular supplementation of cobalamin, but their serum levels were still low-normal at 315 and 428 pg/ml respectively.

It should also be pointed out that four of the five patients (80%) with macrocytic anemia also had low-normal cobalamin levels. Holotranscobalamin and MMA measurements were available for two patients (*n* = 2/5; 40%) and true cobalamin deficiency was ruled out for both. The remaining patient with macrocytic anemia showed no indication of possible cobalamin deficiency with a cobalamin level of 518 pg/ml (folic acid was also in the normal range for all five patients).

### Possible influences on cobalamin metabolism

Serum cobalamin levels below 400 pg/ml (equaling lower normal levels) were measured in 70 patients (*n* = 70/155; 45%) and occurred significantly more often in HHT patients with gastrointestinal involvement (*p* = 0.027) whilst epistaxis severity or other organ involvements did not show such an effect (*p* > 0.05). A total of 50 patients (*n* = 50/155; 32%) had gastrointestinal vascular malformations. Of these, 29 patients (*n* = 29/50; 58%) and only 41 of patients without gastrointestinal involvement (*n* = 41/105; 39%) had cobalamin levels below 400 pg/ml. Cobalamin levels did not differ significantly between patients with treated and untreated gastrointestinal angiodysplasia (*n* = 30/50; 60% with past APC, *n* = 20/50; 40% without, *p* = 0,691).

Almost every fifth patient (*n* = 30/155; 19%) received medication known to be associated with cobalamin deficiency (proton pump inhibitors (PPI)^28,29^: *n* = 25/30; 83%, metformin^30^: *n* = 3/30 10% and both: *n* = 2/30; 7%). 15 patients with HHT (*n* = 15/155; 10%) reported gastrointestinal comorbidities (past gastric bypass surgery: *n* = 1/15; 7% and gastrointestinal inflammatory disease (gastritis with or without ulcers, reflux esophagitis, hepatitis or pancreatitis): *n* = 14/16%). Neither medication (PPI or metformin) nor gastrointestinal comorbidities were significantly associated with cobalamin levels (*p* > 0.05 respectively), even if there was a trend towards lower cobalamin serum levels in patients with gastrointestinal inflammatory diseases at *p* = 0.08.

### Potential symptoms of cobalamin deficiency and nutrition

A total of 43 patients with HHT and cobalamin levels below 400 pg/ml (*n* = 43/70; 61%) could be questioned extensively concerning possible symptoms of cobalamin deficiency (cutaneous, gastrointestinal, hematological and neurological symptoms). The most commonly reported symptoms were shortness of breath (*n* = 29/43, 67%) and tiredness and sleep disturbances (*n* = 28/43; 65%). Most patients reported at least one symptom from each category (*n* = 37/43; 86% cutaneous, *n* = 39/43; 91% gastrointestinal, *n* = 42/43; 98% hematological and *n* = 42/43; 98% neurological symptoms). Paresthesia, falling tendencies, difficulty in concentration and impaired sensitivity are the symptoms most specific for cobalamin deficiency over general anemia or iron deficiency. However, only difficulty with concentration was actually reported by more patients with low or low normal cobalamin levels (*p* = 0.059). None of these symptoms showed correlations to hemoglobin levels. Table 4 provides a detailed overview of the reported symptoms.

**Table 4:** Questionnaire of possible symptoms associated with cobalamin deficiency.

Symptom	Number of positive answers
**At least one cutaneous symptom**	**37 (86%)**
Pallor or irregular skin coloring	20 (47%)
Brittle nails	20 (47%)
Coldness of hands or feet	21 (49%)
**At least one gastrointestinal symptom**	**39 (91%)**
Swelling or irritation of tongue, mucosal irregularities	17 (40%)
Pallor of oral mucosa	5 (12%)
Digestive irregularities (e.g. constipation, diarrhea)	24 (56%)
Unexplainable weight gain or difficulty losing weight	11 (26%)
Comorbidities or anatomical abnormalities	19 (44%)
Recurring gastrointestinal bleeding	5 (12%)
**At least one hematological symptom**	**42 (98%)**
Shortness of breath after little exertion	29 (67%)
Consumption of iron, cobalamin or folic acid supplements	23 (53%)
Need for blood transfusions	18 (42%)
Need for regular intravenous iron substitution	22 (51%)
**At least one neurological symptom**	**42 (98%)**
Paresthesia or irregular sensations in hand and/or feet	25 (58%)
Dizziness or feeling dazed	21 (49%)
Pathological gait, tendency to fall	13 (30%)
Headaches (regularly or even daily)	13 (30%)
Weakness or tiredness	24 (56%)
Sleeping disturbances or unrestful sleep	28 (65%)
Difficulty concentrating or remembering	22 (51%)
Impairment of vision	15 (35%)
Impaired sensibility (touch, vibration)	5 (12%)

In total 43 patients with hereditary hemorrhagic telangiectasia and cobalamin levels below 400 pg / ml were questioned extensively on the presence or absence of each symptom. The number of positive responses and the corresponding percentages for each symptom are provided.

A vegetarian diet was reported in two patients (*n* = 2/43; 5%), one patient out of 43 (2%) ate mostly vegan and another three (*n* = 3/43; 7%) reported low consumption of meat. An average of 2–3 glasses of wine or beer per week was consumed, and about half of the patients (*n* = 22/43; 51%) did not drink alcohol at all, mostly due to increased epistaxis after alcohol consumption. Neither nutrition nor alcohol consumption correlated with cobalamin levels (*p* > 0.05).

### Interaction between cobalamin and iron deficiency

Low cobalamin levels were seen often in both iron deficient patients (*n* = 43/101; 43%) and in patients without iron deficiency (*n* = 27/54; 50%; *p* = 0.675). Both cobalamin levels below 400 pg/ml and overall iron deficiency were present in 43 patients (*n* = 43/155; 28%). Two thirds of these patients were also anemic (*n* = 29/43; 67%; hemoglobin 10.7 ± 2.7 g/dl) which was a significantly higher percentage compared to the rest of the patient population (*n* = 36/112; 32.1%; hemoglobin 12.9 ± 2.2 g/dl; *p* < 0.001). All forms of anemia occurred in these patients with more than half of the patients presenting as microcytic (*n* = 17/29; 59%) and almost a third as normocytic (*n* = 9/29; 31%). A minority of patients had macrocytic anemia (*n* = 3/29; 10%) even in the presence of iron deficiency. As expected, decreased MCV was significantly more common in iron deficient patients (*p* < 0.001). No statistically significant correlation was observed between the presence of iron deficiency and the presence of symptoms most specifically attributed to iron deficiency over general anemia or cobalamin deficiency (skin lesions, brittle nails, paleness of oral mucosa, sensation of burning tongue).

## Discussion

For the first time to our knowledge, our study evaluated cobalamin levels in HHT patients with many patients having low normal and in some cases even insufficient cobalamin levels (similar to the general population). Compared to the general population, true cobalamin deficiency might occur even less often. We could also demonstrate that iron deficiency remains a challenging aspect in patients with HHT.

### Iron deficiency in HHT

All patients with HHT analyzed in this study suffered from epistaxis with mostly moderate and sometimes severe severity according to the ESS, leading to anemia. Two thirds of the patients with low hemoglobin levels had a microcytic anemia which is typical for an iron deficiency anemia^31,32^. However, MCV was also often normal and sometimes even elevated. This illustrates the need for additional laboratory parameters such as ferritin, C reactive protein and soluble transferrin receptor to evaluate HHT patients’ iron status correctly^33^ as conducted in this patient cohort.

At the point of study inclusion, only a third of patients with an iron deficient status and less than half of the patients with lowered hemoglobin levels received iron orally and / or intravenously. More than a third of the HHT patients had already received blood transfusions and 10% of these patients were tested positive for blood group antibodies. These are at risk for severe complications in future transfusions. Early adjustment of iron substitution could possibly have raised hemoglobin levels sufficiently in order to avoid more invasive treatment options such as blood transfusions. Despite this, more than two thirds of the HHT patients who regularly used iron substitutions were still classified as iron deficient assuming an insufficient intake. Patients reported gastrointestinal side effects and lack of awareness as possible reasons for this. Recently, it was found that intermittent iron substitution of only once up to three times a week on non-consecutive days have similar effects on hemoglobin levels but less gastrointestinal adverse effects compared to the daily intake^34,35^ which had often lead to therapy discontinuation before. Intravenous iron substitution might be an alternative or additive therapeutic option for those patients who after optimizing their oral iron intake still experience side effects or an insufficient increase of their iron parameters. However, it needs to be administered by a physician. This can be problematic for some patients when their (most often general) physicians are not experienced in the treatment of chronic anemia in HHT patients or might not be aware of the particularities of the patients’ situation with recurring episodes of bleeding in various intensity. Many patients also described difficulties in obtaining prescriptions for iron preparations, tranexamic acid tablets or referrals to hematologists. While the exact reasons for this were not clear, there are some possible explanations: Especially in the past, more undesirable side effects (especially allergic or cardiovascular reactions) had been described for intravenous iron substitution compared to oral therapy^36^. Moreover, iron infusions are significantly more expensive compared to oral iron substances, which also might negatively affect funding particularly for general practitioners in Germany. According to the recommendations of the European Reference Network (ERN)^37^, at our HHT center it is offered to check for anemia and iron parameters at each visit and we always give patients written advice to monitor hemoglobin and iron status regularly as well as to initiate or adjust iron substitution as needed.

### Interpretation of cobalamin levels in HHT patients

Almost half of the patients with HHT showed low-normal levels of cobalamin especially when hemoglobin levels were below the norm. Low-normal cobalamin levels are also frequently observed in the general population. Large cohort studies from the United States found median cobalamin levels of around 400 pg/ml whilst levels below 300 pg/ml were measured for 40% of more than 5000 patients in a Colombian study. However, anemia or macrocytosis were observed far less frequently^38^. In line with this, in our study neither MCV, MCH nor MCHC correlated to cobalamin levels at all. We attributed this to the high concurrence of iron deficiency which can mask cobalamin deficiency as macrocytosis is often not observed^39^.

The absolute cobalamin levels are affected by many factors (e.g. age, special diets, toxic substances, chronic liver diseases, myeloproliferative neoplasms, anti-intrinsic factor antibodies^40^, oxidative stress^41^). However, in our study neither alcohol consumption nor chronic diseases showed any effect on cobalamin levels. In the general population, cobalamin deficiency (cobalamin levels below the respective normal range or deficiency diagnosed by further evaluation of holotranscobalamin and MMA) is found in 5–7% of young adults^42^ and far more commonly (20–25%) in patients aged 60 years and above^40,43^ as gastric tissues which release intrinsic factor atrophy with age^7^. Since HHT is a rare condition with symptoms often increasing with age^1,32^, a diagnostic latency is described and HHT is often diagnosed later in life^44^. Accordingly, more than one third of our patients were aged 60 years or above, hence belonging to the high-risk group for cobalamin deficiency. However, cobalamin levels below the reference range were only seen in less than 1% of our study cohort which is even less than found in the general populations described above. It is questionable, if the nutritional habits of these populations correspond to the current German general population. Therefore, we restrict our conclusion to the statement that a cobalamin deficiency in HHT is not diagnosed more frequently than in the general population.

Nutrition is also known to influence cobalamin levels when consumption of animal products is restricted. However, neither vegan nor vegetarian patients with HHT showed cobalamin levels diverging significantly from the rest of the study population. The percentage of patients with HHT who did not eat animal products did not differ significantly from the one found in the German population^45,46^.

Low-normal cobalamin levels (below 400 pg/ml) were found significantly more often in patients with HHT and gastrointestinal involvement compared to those patients without gastrointestinal angiodysplasia. Hemoglobin levels were also significantly lower in patients with gastrointestinal involvement. A potential hypothesis might be that cobalamin uptake could be impaired in these patients as gastrointestinal angiodysplasia reduce the overall mucosal tissue area. They could also lead to recurrent bleedings which exacerbate anemia, iron and cobalamin deficiency. Coagulation therapy of gastrointestinal lesions, however, had no significant effect on cobalamin levels.

### Potential clinical relevance of cobalamin deficiency

Clinical relevance of cobalamin deficiency was assessed through a self-reported questionnaire. Even though only very few patients with HHT suffered from a true cobalamin deficiency (having been ruled out by analysis of holotranscobalamin and MMA), a large part of patients with cobalamin levels below 400 pg/ml reported a variety of symptoms typically associated with cobalamin deficiency. However, almost all of these symptoms were non-specific for cobalamin deficiency. Especially hematologic symptoms such as the general feelings of weakness and dizziness could just as easily be caused by concomitant iron deficiency anemia or in part even by surrounding factors, e.g. sleep deprivation or stress. It has also already been reported that neurological symptoms such as the restless legs syndrome due to an iron deficiency can present in patients with HHT^47^. One third of the patients with cobalamin levels below 400 pg/ml could be questioned extensively. On average, they reported nine symptoms associated with cobalamin deficiency even though their iron and hemoglobin levels were normal. However, both cobalamin deficiency and resulting high levels of homocysteine are associated with neurological degeneration and cognitive impairment^48^ which could be easily and cost-effectively remedied with sufficient substitution of cobalamin (either by oral or intramuscular replacement)^12,20,49,50^. Normalization of cobalamin levels and relating laboratory parameters should occur within two months^50^. This study showed that low-normal Cobalamin levels were found often in patients with HHT; however, the prevalence of a true cobalamin deficiency and lower normal cobalamin concentrations does not seem to be increased in HHT compared to the general population and the clinical impact remains unclear. Additionally, even in the few cases of cobalamin deficiency we could not observe relevant symptoms clearly associated with this deficiency. However, a positive effect of high cobalamin levels on cancer development is discussed^51^. Thus, in cases of symptomatic patients where concurrent reasons (esp. iron deficiency) have been ruled out, cobalamin might be measured and in case of deficiency substituted. Cobalamin supplementation in patients with low-normal cobalamin levels (especially in those patients with symptoms mentioned above) could be discussed individually.

### Combination of cobalamin and iron deficiency

Approximately 28% of the study population exhibited concurrent iron deficiency and low or low-normal cobalamin levels; however, no specific clinical symptom was found to be significantly associated with either deficiency. Patients in this subgroup were significantly more likely to suffer from anemia compared to patients with only one or none of these characteristics. MCV is most commonly used as the first parameter for evaluation of both possible deficiencies, but proved to be unreliable especially in this patient population. Most patients were microcytic even though cobalamin levels were low or low-normal. On the other hand, even macrocytosis was observed in three patients of this group (*n* = 3/43; 7.0%). Another interesting finding was that 38% of iron deficient patients had normal MCV (*n* = 33/87) whereas 36% of patients with microcytosis (*n* = 22/61) did not display overall iron deficiency. This accentuates the challenge of diagnosing the correct type of anemia patients with HHT and providing individual treatment recommendations respectively. Additional blood analysis (e.g. complete iron parameter status and at least MMA) might be often crucial for an accurate assessment of possible deficiencies.

### Study limitations

The main limitations of this study are its partially retrospective nature and reliance on patients’ recall of symptoms, which are in part highly subjective to begin with. Although a laboratory workup is recommended in all patients with HHT each time they visit our clinic, not all patients agreed to it. Noticing low-normal cobalamin levels in our routine work up, we started to measure holotranscobalamin and methyl malonic acid. Therefore, in patients who were included in this study before spring 2022 we did not receive these additional measurements.

So far, no validated questionnaire to investigate clinical symptoms of cobalamin deficiency exists. Reported symptoms of cobalamin deficiency are not very specific and could be also explained by other causes such as iron deficiency, stress or lack of sleep. However, in our study, patients with no signs of iron deficiency but low-normal cobalamin levels reported clinical symptoms. These patients were advised to substitute cobalamin after consultation with their general practitioners. Since both cobalamin and iron requirements vary among the study population and different dosages have been discussed and recommended in the literature^12,38,47^, patients did not follow one set plan for either supplementation. Due to the cross-sectional design of the study, we were unable to obtain data on the success of substitution. Further studies should evaluate whether the monitoring of cobalamin parameters and the adequate substitution of cobalamin alleviate symptoms and therefore improve patients’ quality of life.

Additional laboratory workup (i.e., measurement of holotranscobalamin and methyl malonic acid) had to be performed in external laboratories even for our university hospital, being a tertiary care center and might be difficult to obtain for smaller clinics, complicating an accurate assessment of cobalamin (and iron) status. Pernicious anemia (the presence of autoimmune antibodies against gastric parietal cells or intrinsic factor) is a differential diagnosis for cobalamin deficiency^16^. It could not be excluded with absolute certainty for the seven patients we identified as cobalamin deficient, since the Schilling test is currently not available due to cessation of production.

## Conclusion

Our data suggests that patients with HHT suffer slightly less often from true cobalamin deficiencies compared to the normal population. However, low-normal cobalamin levels were frequently observed in these patients (like in the general population) for which the clinical relevance remains unclear. Iron deficiency was frequently diagnosed in HHT and could have masked a beginning cobalamin deficiency. Therefore, laboratory workups of cobalamin levels and additional parameters such as MMA and holotranscobalamin - if low-normal cobalamin levels are found – could be considered to detect a beginning cobalamin deficiency. We consider this approach in symptomatic cases in which other causes (esp. iron deficiency) have been ruled out. This could be done especially if patients are anemic, symptoms associated with cobalamin deficiency are present or known risk factors for cobalamin deficiency (medication with proton pump inhibitors or metformin, gastrointestinal inflammation, high bleeding or past interventions) exist.

## Supplementary Information

Below is the link to the electronic supplementary material.

Supplementary Material 1

## Data Availability

The datasets generated and/or analysed during the current study are not publicly available since they contain pseudonymized patient data but are available from the corresponding author on reasonable request.
