# Supplementary material for: Cobalamin and iron deficiency still presents a challenge in hereditary hemorrhagic telangiectasia
Source: Sci Rep. 2025 Aug 4;15:28463. doi: 10.1038/s41598-025-13911-6 (PMC12321984; doi:10.1038/s41598-025-13911-6)
Supplement: Supplementary file 1 — Supplementary Material 1 [file 41598_2025_13911_MOESM1_ESM.docx]

**List of Abbreviations**

HHT - hereditary hemorrhagic telangiectasia

Hb - hemoglobin

MCV - mean corpuscular volume

CRP - C-reactive protein

ESS - epistaxis severity score

m - mean

SD - standard deviation

MMA - methyl malonic acid

PPI - proton pump inhibitors

MCH - mean corpuscular hemoglobin

MCHC - mean corpuscular hemoglobin concentration

GOT - glutamate-oxalacetate-transaminase

GPT - glutamate-pyruvate-transaminase

i.v. - intravenous

**Appendix**

Symptoms in patients presenting with cobalamin levels <400 pg/ml

Patients were asked whether they experienced the following symptoms with responses limited to “yes” or “no”. They were also asked to provide current information on nutritional habits, alcohol consumption, comorbidities (especially diabetes, gastrointestinal diseases, autoimmune diseases, hematological diseases), and medication (especially proton pump inhibitors, metformin, vitamins or other supplements).

1. Cutaneous manifestations
   1. Pallor or irregular skin coloring
   2. Brittle nails
   3. Coldness of hand or feet
2. Gastrointestinal manifestations
   1. Swelling or irritation of tongue, mucosal irregularities
   2. Pallor of oral mucosa
   3. Digestive irregularities (e.g. constipation, diarrhea)
   4. Unexplainable weight gain or difficulty losing weight
   5. Comorbidities or anatomical abnormalities (e.g. past coagulation treatments or operations, inflammatory diseases)
   6. Recurring gastrointestinal bleeding
3. Hematological manifestations
   1. Shortness of breath after little exertion
   2. Consumption of iron, cobalamin or folic acid supplements
   3. Need for blood transfusions
   4. Need for regular intravenous iron substitution
4. Neurological manifestations
   1. Paranesthesia or irregular sensations in hand and/or feet
   2. Dizziness or feeling dazed
   3. Pathological gait, tendency to fall
   4. Headaches (regularly or even daily)
   5. Weakness or tiredness
   6. Sleeping disturbances or unrestful sleep
   7. Difficulty concentrating or remembering
   8. Impairment of vision
   9. Impaired sensibility (touch, vibration)
5. Additional free text to further elaborate on symptoms if needed
